# Conformation of dehydropentapeptides containing four achiral amino acid residues – controlling the role of L-valine

**DOI:** 10.3762/bjoc.10.58

**Published:** 2014-03-14

**Authors:** Michał Jewgiński, Joanna Krzciuk-Gula, Maciej Makowski, Rafał Latajka, Paweł Kafarski

**Affiliations:** 1 Department of Bioorganic Chemistry, Faculty of Chemistry, Wrocław University of Technology, Wybrzeze Wyspianskiego 27, 50-370 Wroclaw, Poland; 2 Faculty of Chemistry, University of Opole, Oleska 48, 45-052 Opole, Poland

**Keywords:** conformation, dehydroalanine, dehydropeptide, dehydrophenylalanine, NMR

## Abstract

Structural studies of pentapeptides containing an achiral block, built from two dehydroamino acid residues (Δ^Z^Phe and ΔAla) and two glycines, as well as one chiral L-Val residue were performed using NMR spectroscopy. The key role of the L-Val residue in the generation of the secondary structure of peptides is discussed. The obtained results suggest that the strongest influence on the conformation of peptides arises from a valine residue inserted at the C-terminal position. The most ordered conformation was found for peptide Boc-Gly-ΔAla-Gly-Δ^Z^Phe-Val-OMe (**3**), which adopts a right-handed helical conformation.

## Introduction

Structurally modified peptides are becoming increasingly interesting as potential substances with pharmacological effects [1]. Dehydropeptides are one representative of this group of compounds. These peptidomimetics and natural peptides are characterized by a double bond between the Cα and Cβ atoms [2]. This modification entails a lot of structural consequences in the conformation of the peptides. The presence of a Cα–Cβ double bond together with two flanking peptide bonds leads to the coupling of π-electrons. This results in a planar structure of the dehydroamino acid moiety [3]. The stiffening of this part of the peptide restricts the allowed conformational space not only at the dehydroamino acid side chain but also has a significant effect on the conformation of the whole dehydropeptide [4–7]. It is noteworthy that the introduction of dehydroamino acid residues has a strong influence on the conformational preference of the peptide chain, irrespective of other constraints. The introduction of (Z)-dehydrophenylalanine stabilizes a β-turn conformation even in the case of short peptides [8–10]. This influence is stronger for peptides with a longer main chain, for which a 3_10_-helical conformation could be observed [8,11–13]. Peptides containing a dehydroalanine residue usually have an inverse γ-turn conformation [7,14–15].

Another important issue of the determination of the secondary structure and properties of peptides is the *Z/E*-isomerism of dehydroamino acids. This phenomenon can be critical for their biological activity [16–18] and for the secondary structure of the peptide chain. Generally, the (Z)-isomers are thermodynamically more stable and easier to obtain. As a consequence, the (Z)-isomers are much more common than the (E)-counterpart [19].

Dehydroamino acids contribute to a catalytic role in the active sites of some enzymes [20–22]. Additionally, the presence of these dehydrogenated analogues of amino acids is crucial for the biological activity of various peptide antibiotics [23], e.g., nisin (used as a food preservative [24]), epidermin (active against Gram positive bacteria [25]) and viomycin (used to fight infections of Mycobacterium tuberculosis [26]). As shown by Chauhan and co-workers, structure–activity characteristics of the bioactive peptides could be studied by the introduction of unsaturated analogues of amino acids into the main sequence. This strategy could lead to novel analogues with a higher activity [8].

**Figure 1:**
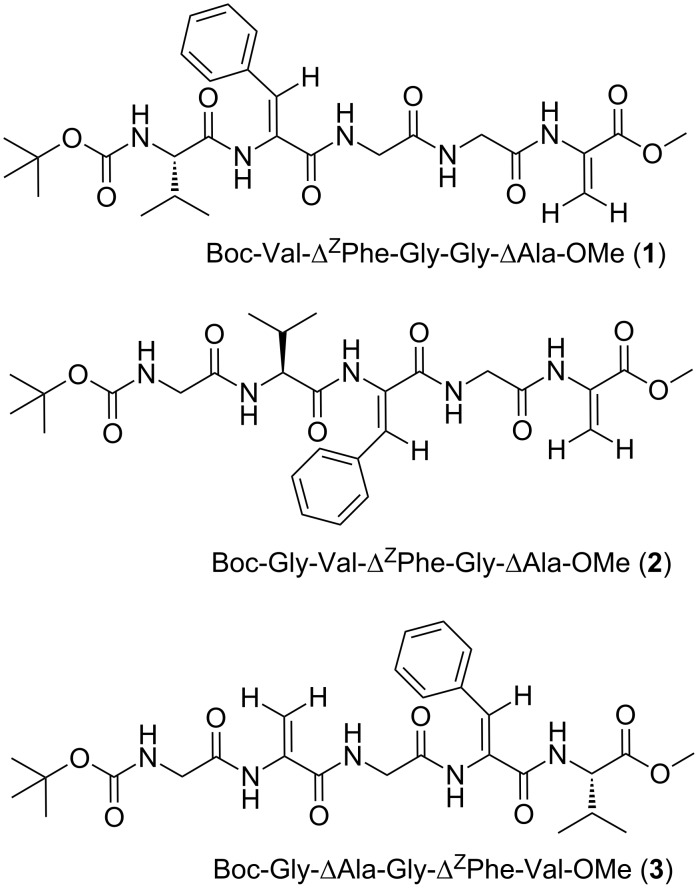
Sequences of the investigated dehydropeptides.

In our previous studies, we investigated pentapeptides containing two dehydroamino acid residues (Δ^Z^Phe and Δ^E^Phe). The results suggested that they prefer bent conformations and existed in two predominant conformations [9]. In the current work, we investigated the influence of the introduction of a Val residue in positions 1, 2 or 5 of the peptide chain on the conformation of dehydropentapeptides containing two dehydroamino acid residues and two additional achiral residues (Figure 1). Structural investigations of dehydropeptides containing an L-valine residue beside a block of achiral amino acid residues were published. Such blocks are built from either three consecutive Δ^Z^Phe [27] or from Aib residues and one Δ^Z^Phe residue [28]. These publications show the crucial role of a L-Val present in the N- or C-terminal position for the forming of a secondary structure. To the best of our knowledge, there is no publication on the conformational preference of dehydropeptides containing both dehydroalanine and dehydrophenylalanine residues. Therefore, we decided to check the influence of the introduction of a L-Val residue at different positions of dehydropeptides containing dehydroalanine and dehydrophenylalanine residues as well as two highly flexible glycine residues. We decided that the determination of the correlation between the position of the L-Val residue in the peptide sequence and its conformation would be important and crucial for the rational design of the sequence of the peptidomimetic foldamers.

## Results and Discussion

### Structural and conformational studies

NMR investigations were carried out to determine the structures and the conformational preferences of the three dehydropentapeptides shown in Figure 1.

#### NMR spectroscopy

The combination of standard 1D and 2D NMR experiments, especially TOCSY, ^1^H,^13^C HSQC and ^1^H,^13^C HMBC, formed the basis of structural studies of the investigated dehydropeptides.

Next, we determined the impact of an increased temperature on the amide proton chemical shift. The characteristic changes of the chemical shifts of amide protons may indicate the existence of hydrogen bonds between amide protons and oxygen atoms in the main chain. The value of the temperature factor dδ/dT is the most revealing parameter to draw conclusions regarding the presence of a hydrogen bond. A value of the temperature factor lower than 4.0 [ppb/K] indicates the presence of a hydrogen bond [29]. The obtained temperature factors for the studied peptide are shown in Table 1.

**Table 1:** Temperature dependence of the chemical shifts of amide protons of the investigated peptides – temperature coefficient dδ/dT [ppb/K].

peptide					

**1**	HN Val(1)	HN Δ^z^Phe(2)	HN Gly(3)	HN Gly(4)	HN ΔAla(5)
	**1.1**	14.0	6.2	11.0	11.1

**2**	HN Gly(1)	HN Val(2)	HN Δ^z^Phe(3)	HN Gly(4)	HN ΔAla(5)
	11.0	7.3	9.5	6.1	7.3

**3**	HN Gly(1)	HN ΔAla(2)	HN Gly(3)	HN Δ^z^Phe(4)	HN Val(5)
	13.1	**4.9**	10.9	10.2	11.7

Generally, the results obtained from temperature experiments show that structures of the investigated peptides are not stabilized by intramolecular hydrogen bonds. Only in the case of peptide **1***,* the value of the temperature coefficient of the amide proton of the valine residue in the first position indicates that it is involved in an intramolecular hydrogen bond. In the case of peptide **3**, the temperature factor of the amide proton of ΔAla(2) is slightly higher than the threshold indicating the presence of hydrogen bonding. Such a value could suggest that the amide proton of ΔAla(2) is at least partially resistant to the influence of the temperature and may create a weak hydrogen bond.

While the results of the temperature experiments give important structural information, the most insightful data about the conformational preferences was obtained from 2D ROESY experiments. Based on the intensities of the assignment of cross and diagonal peaks in the ROESY spectra, hydrogen–hydrogen distances were calculated [30]. In addition of distance constraints we also used dihedral angle constraints during the structural calculations. These structural constraints, imposed on the backbone φ dihedral angle, were determined on the basis of the vicinal coupling constants between the amide proton and the alpha proton [31]. Information about the number of structural constraints applied during calculation is summarized in Table 2. Based on the determined structural constraints, the conformational preferences of the investigated peptides were defined by using the XPLOR-NIH [32] program. The average values obtained for dihedral angles after the clustering of received conformers are presented in Table 3*.*

**Table 2:** Number of structural constraints used during calculations.

	NOE constraints			

peptide	Strong (1.8–2.5 Å)	Medium (1.8–3.5 Å)	Weak (1.8–5.0 Å)	^3^*J*_HN-HA_

**1**	3	14	10	1
**2**	7	13	40	1
**3**	7	16	20	1

**Table 3:** Average values of dihedral angles [°] obtained on base of XPLOR-NIH calculations.

Peptide	Size of cluster	Dihedral angle [°] with standard deviation								
		φ_1_	ψ_1_	φ_2_	ψ_2_	φ_3_	ψ_3_	φ_4_	ψ_4_	φ_5_

**1a**	15.5%	−88 ± 4	19 ± 12	79 ± 15	69 ± 5	−91 ± 22	−8 ± 28	112 ± 44	NE	NE
**1b**	13.0%	−79 ± 10	−15 ± 16	−59 ± 20	−76 ± 5	−70 ± 16	−27 ± 21	123 ± 39	NE	NE
**2a**	25.4%	NE	NE	NE	−63 ± 17	−35 ± 16	79 ± 4	77 ± 5	−3 ± 9	43 ± 9
**2b**	22.1%	NE	NE	NE	−72 ± 7	132 ± 13	−76 ± 3	−64 ± 8	−9 ± 22	20 ± 32
**3**	~100%	NE	NE	−80 ± 11	−74 ± 5	−65 ± 7	−49 ± 9	−72 ± 10	−75 ± 11	NE

NE – Not estimated during cluster analysis.

In the case of peptides **1** and **2** the results of the calculations suggest that peptides could adopt two types of bent conformations, whereas for peptide **3** only one well-defined bent conformation could be found. Selected φ and ψ dihedral angles were excluded during clustering analysis for all investigated peptides. When we tried to group the obtained conformers according to all torsion angles of the main chain, the analysis did not lead to any conformational cluster at all or showed very few conformational clusters.

**Figure 2:**
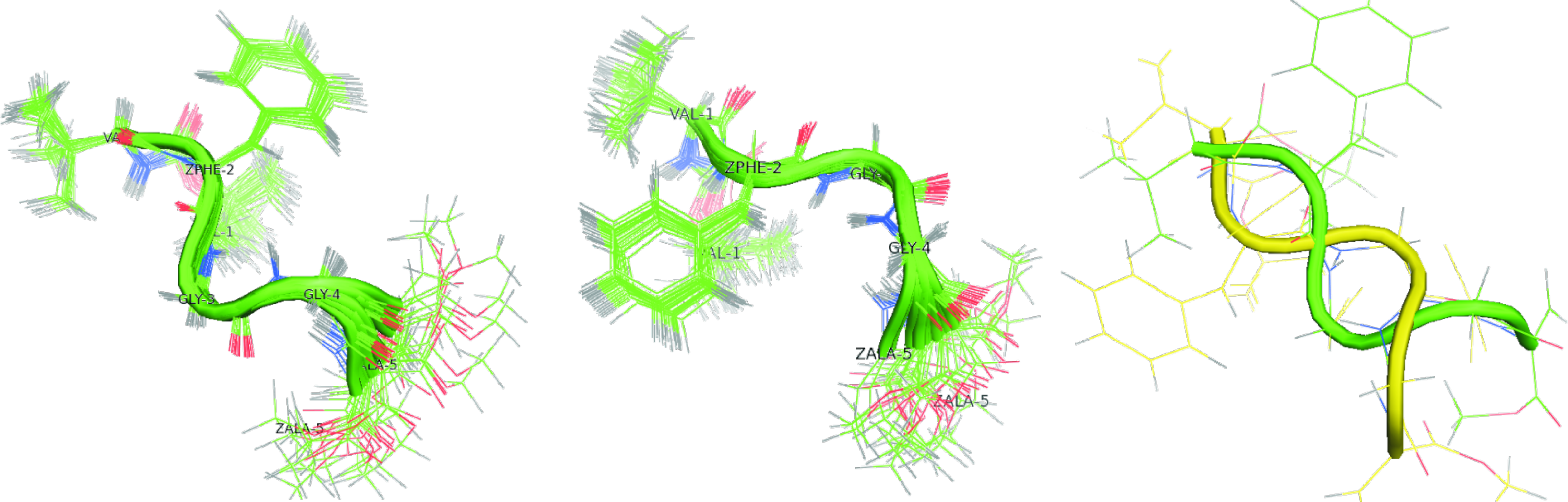
The most stable conformation of peptide **1** proposed based on XPLOR-NIH calculations. Conformation **1a** (left), conformation **1b** (middle) and a superposition of both conformations (right).

The values of the standard deviation of the main chain torsion angle observed for peptide **1** hint at a highly flexible block of two glycine residues Gly(3)–Gly(4) and an instability of this part of the peptide chain. Strong divergence of the torsion angles values, which are observed for typical secondary structures, render it difficult to clearly determine the secondary structure of peptide **1**. However, the obtained mean values of the torsion angles suggest an intermediate structure between β-turn type I and type II, which is localized on the N-terminal part of the peptide. The values of the dihedral angles indicate that peptide **1** could display two types of a bent conformation in DMSO solution with an opposite orientation of turns (see Figure 2).

**Figure 3:**
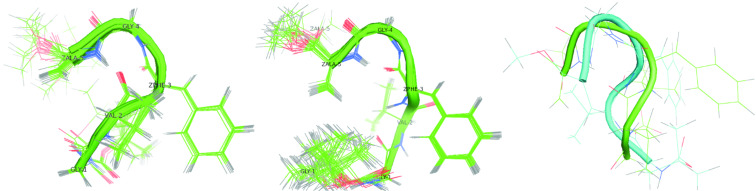
Proposal of the most stable conformation of peptide **2** based on XPLOR-NIH calculation. Conformation **2a** (left), conformation **2b** (middle) and a superposition of both conformation (right).

As in the case of the peptide **1**, our analysis of the obtained structures for peptide **2** leads to two clusters. The smaller values of the standard deviation of the dihedral angles (see Table 3) suggest that the conformations of peptide **2** are much better defined. Possible types of β-turn conformations could be easily distinguished by the use of NMR techniques based on the NOEs effects [33]. Characteristically strong NOE signals between the alpha proton from *i* and the amide proton from the consecutive *i+1* residues indicate the type II β-turn [34]. This type of secondary structure is also confirmed by weak NOE signals from interactions between the amide protons of consecutive residues [35]. The presence of two consecutive β-turns type II are suggested by the observed short distances between C^α^H Gly(1) and HN Val(2) and between Hα Val(2) and HN Δ^Z^Phe(3), respectively. Additional evidence is provided by the long distances (~4.5 Å) between amide protons of Gly(1) and Val(2) as well as Val(2) and Δ^Z^Phe(3) (see Table S7 in Supporting Information File 1). This observation seems to be confirmed by the result of the XPLOR calculation. Figure 3 shows two consecutive bents for both clusters **2a** and **2b**, which were found after clustering the results of the XPLOR calculations. However, the estimated mean values of the φ and ψ torsion angles (see Table 3) do not allow for clearly identifying the type of secondary structure.

**Figure 4:**
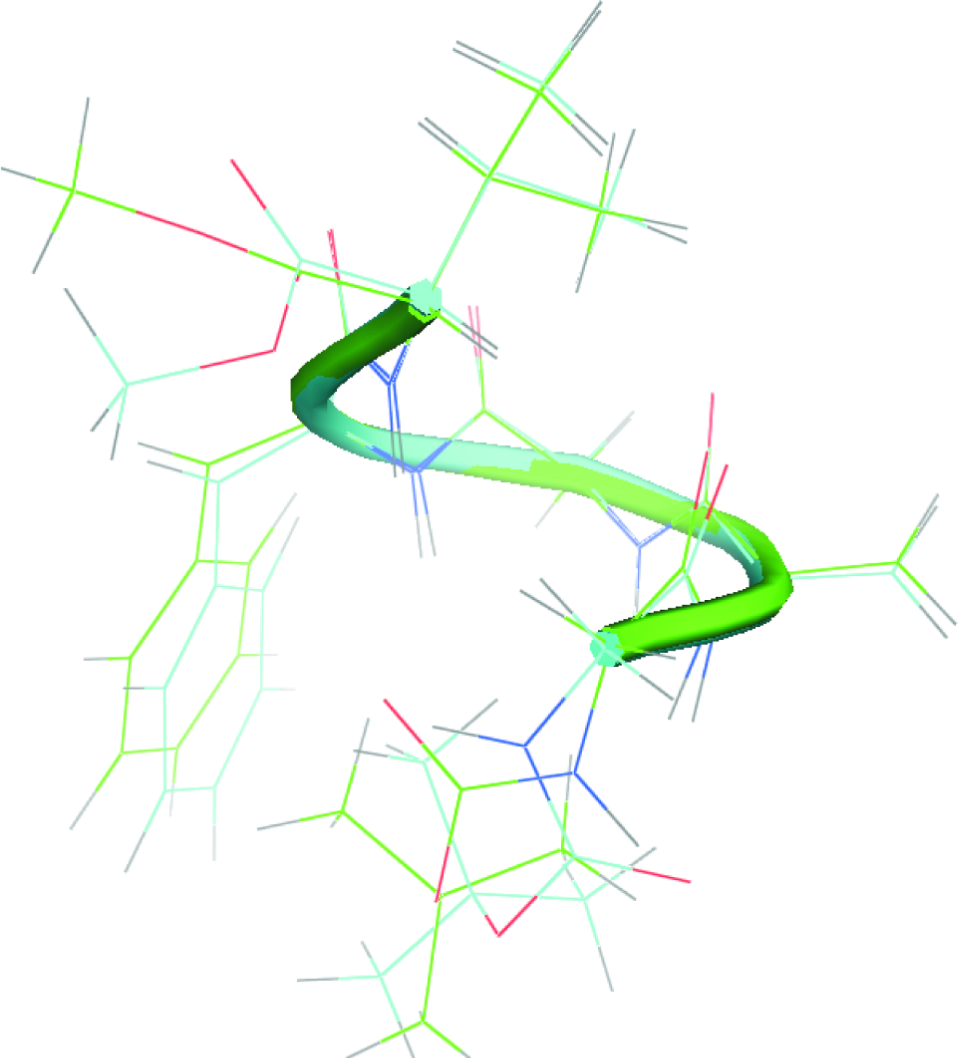
Proposal of the most stable conformation of peptide **3** based on XPLOR-NIH calculations.

Although we observed the largest number of structural constraints for peptide **2** (see Table 2), we still encountered difficulties in the unambiguous determination of the preferred conformation. The reason for this may be the simultaneous coexistence of two types of conformations in solution – we observed NOE signals from both of them. In the case of peptide **3**, structural calculations based on a much smaller number of structural constraints led to only one well-defined conformation. As shown in Figure 4 peptide **3** has a helical conformation. Both α- and 3_10_-helix give a similar vicinity of the backbone protons of nonconsecutive residues between Hα*_i_*–HN*_i+2_* and Hα*_i_*–HN*_i+3_* [35]. Inai and Hirabayashi showed that the presence of NOE signals between Hα*_i_*–HN*_i+4_* could be used to verify the presence of an α-helix [36]. In case of peptide **3** the observation of NOE signals Hα*_i_*–HN*_i+2_* and Hα*_i_*–HN*_i+4_* between Hα[Gly3]-HN[Val5] and Hα[Gly1]-HN[Val5], can be interpreted as a sign of an α-helical conformation. Furthermore, the analysis of the representative values of the torsion angles of the main chain of the obtained conformational cluster indicates that peptide **3** exhibits a right-handed helix [28]. Only such a bent structure with a flexible N-terminal part could explain the strong NOE signals between the methyl residue of the Boc group and the side chain of both the dehydroamino acids and NOE signals between the alpha protons of Gly(1) and Val(5) (see Table S9 in Supporting Information File 1). This result is inconsistent with previously published conformational preferences of dehydropeptides containing only one L-amino acid ester at the C-terminal position [37]. Toniolo and co-workers described that the insertion of an ester at the chiral residue of the dehydropeptide sequence induces left-handed helical structures. However, in the case of peptide **3**, the analysis of the torsion angle values of the main chain clearly indicates that the insertion of one chiral L-Val methyl ester at the C-terminal position of the peptide induced a right-handed helical structure. We assume that the helix adopted by peptide **3** is far from the ideal helical conformation, so that an unfavorable O∙∙∙O interaction does not occur.

As could be seen from our previous investigations, an increase in solvent polarity promotes more ordered conformations of hexapeptides containing two dehydrophenylalanine residues [38]. On the other hand, Inai and co-workers showed that in the case of dehydropeptides containing only one chiral L-residue in the N-terminal position of the peptide a left-handed helical conformation is adopted regardless of the solvent polarity [39], but when a chiral residue occupied the second position of the peptide, the screw sense of the helix depends on the type of solvent [40].

## Conclusion

The conformational studies in solution performed by NMR spectroscopy show that all investigated dehydropentapeptides exhibit an ordered conformation. The level of ordering depends on the location of the chiral residue of the single amino acid in the peptide sequence.

I) If the L-Val residue occupies the N-terminal position and two dehydroamino acids are separated by two consecutive glycine residues, pentapeptide **1** exists in two different conformations, with a relatively high flexibility of the main chain. Such a high flexibility was previously observed for peptides containing Δaa-Gly-Gly-Δaa sequences [41].

II) If the L-Val residue is introduced in the second position and dehydroamino acid residues are separated by one glycine residue, dehydropeptide **2** displays two sequential β-turns. Furthermore, observed conformations are much more stable than found for peptide **1**.

III) Introduction of the L-Val residue into the C-terminal position generates the helical conformation of peptide **3**. Despite the fact that peptide **3** contains an ester form at the C-terminal position a right-handed helix is observed. This observation is opposite to the previously described preferences of peptides containing an L-amino acid ester in the C-terminal position [37]. Furthermore, peptide **3** has the highest stability of the main chain.

## Experimental

### General

Materials were obtained from commercial suppliers (Sigma-Aldrich, Fluka, Merck, Armar) and used without purification unless otherwise stated. Column chromatography was performed on silica gel H60 (70–230 mesh). The complete assignment of ^1^H and ^13^C chemical shift can be found in the Supporting Information File 1.

### Synthesis

Investigated peptides were synthesized in solution by the 2 + 3 method following the mixed-anhydride procedure, similar to methods already described [42–46].

**Peptide Boc-Val-Δ^Z^Phe-Gly-Gly-ΔAla-OMe (1):** Obtained in 76% yield by crystallization from ethyl acetate/methanol/hexane mixture. Mp 221–223 °C. Elementary analysis: anal. calcd: C, 57.95; H,6.66; N,12.51; found: C, 57.81; H, 6.81; N, 12.44.

**Peptide Boc-Gly-Val-Δ^Z^Phe-Gly-ΔAla-OMe (2):** Obtained in 70% yield by crystallization from ethyl acetate/hexane mixture. Mp 189–192 °C dec. Elementary analysis: anal. calcd: C, 57.95; H, 6.66; N, 12.51; found, C, 57.78; H, 6.87; N, 12.40.

**Peptide Boc-Gly-ΔAla-Gly-Δ^Z^Phe-Val-OMe (3):** Obtained in 79% yield by crystallization from ethyl acetate/diethyl ether/hexane mixture. Mp 103–107 °C. Elementary analysis: anal. calcd: C, 57.95; H, 6.66; N; 12.51; found, C, 57.75; H, 6.88; N, 12.38.

### NMR spectroscopy

NMR spectra were recorded on a Bruker Avance II 600 MHz spectrometer in deuterated DMSO-*d*_6_ solution at 298 K. A 10 mM peptide solution was prepared for all measurements. Proton and carbon chemical shifts are given in relation to Si(CH_3_)_4_. The assignment and integration of NMR signals were carried out with Bruker Topspin and SPARKY [47] software. In case of 2D ROESY spectra the separation between the two germinal β protons of dehydroalanine was used as a reference in a distance calculation. Furthermore, calculated distances were corrected by using a correction factor, as described by Ämmälahti et al. [30]. Obtained distances were divided into three groups of constraints (strong: 1.8–2.5 Å, medium: 1.8–3.5 Å, weak: 1.8–5.0 Å) depending on the interatomic distance. The vicinal coupling constant ^3^*J*_HNHA_ was determined based on 1D ^1^H NMR spectra and then used as a dihedral angle constraint. A complete list of the used constraints can be found in Supporting Information File 1. Conformational calculations were carried out by using XPLOR-NIH v2.31 and PARRALLHDG force field [32]. The calculation was based on the topology of building blocks and structural constraints derived from NMR experiments. The investigated peptides contain a non-naturally occurring amino acid residue. This building block was not present in the selected force field, so that it was necessary to build this residue. For every dehydropeptide the determined structural constraints were employed to calculate 1000 conformers by using the XPLOR-NIH [32] program. Structural preferences of the studied peptide were determined on the basis of 20% of the lowest energy conformers (200 conformers). Analysis of the obtained conformations and their clustering were done with the GROMOS analyzing tools.

## Supporting Information

File 1 Supplementary material.
