# Supplementary material for: Conformation of dehydropentapeptides containing four achiral amino acid residues – controlling the role of L-valine
Source: Beilstein J Org Chem. 2014 Mar 14;10:660–6. doi: 10.3762/bjoc.10.58 (PMC3999861; doi:10.3762/bjoc.10.58)
Supplement: File 1 — Supplementary material. [file Beilstein_J_Org_Chem-10-660-s001.pdf]

Supporting Information

for

Conformation of dehydropentapeptides containing four achiral amino acid residues – controlling the role of L-valine

Michał Jewgiński[1]*, Joanna Krzciuk-Gula[1], Maciej Makowski[2], Rafał Latajka[1] and Paweł Kafarski[1]

Address: [1]Department of Bioorganic Chemistry, Faculty of Chemistry, Wrocław University of Technology, Wybrzeze Wyspianskiego 27, 50-370 Wroclaw, Poland and [2]Faculty of Chemistry, University of Opole, Oleska 48, 45-052 Opole, Poland

Email: Michał Jewgiński - michal.jewginski@pwr.wroc.pl

* Corresponding author

Supplementary material

Table S1: ^{1}H NMR (TMS) of Boc-**Val**-*Δ*^{Z}*Phe*-Gly-Gly-*ΔAla*-OMe (**1**)

Group	Atom	Chemical shift [ppm]
**Boc**	CH_3	1.42
**Val[1]**	HN	6.99
	H^{α}	3.92
	H^{β}	2.00
	H^{γ}	0.89, 0.91
**Δ^{Z}Phe[2]**	HN	9.77
	H^{β}	7.03
	H^{δ}	7.62
	H^{ε}	7.38
	H^{ζ}	7.35
**Gly [3]**	HN	8.33
	H^{α}	3.82
**Gly [4]**	HN	8.12
	H^{α}	3.92
**ΔAla[5]**	HN	9.30
	$H^{\beta1}$	5.73
	$H^{\beta2}$	6.23
**OMe**	CH_3	3.34

Table S2: ^{13}C NMR (TMS) of Boc-**Val**-*Δ*^{Z}*Phe*-Gly-Gly-*ΔAla*-OMe (**1**)

Group	Atom	Chemical shift [ppm]
**Boc**	CH_3	28.64
	C	78.94
	C(O)	156.42
**Val [1]**	C^{α}	60.56
	C^{β}	30.14
	C^{γ}	18.90, 19.80
	C(O)	172.66
**Δ^{Z}Phe[2]**	C^{α}	134.33
	C^{β}	127.79

	C^{γ}	134.30
	C^{δ}	130.11
	C^{ε}	128.97
	C^{ζ}	128.89
	C(O)	166.08
**Gly [3]**	C^{α}	43.16
	C(O)	169.96
**Gly [4]**	C^{α}	43.22
	C(O)	168.82
**ΔAla[5]**	C^{α}	132.87
	C^{β}	109.76
	C(O)	164,25
**OMe**	CH_3	53,13

Table S3: ^{1}H NMR (TMS) of Boc-Gly-**Val**-*Δ^{Z}Phe*-Gly-*ΔAla*-OMe (**2**)

Group	Atom	Chemical shift [ppm]
**Boc**	CH_3	1.38
**Gly[1]**	HN	7.00
	H^{α}	3.64
**Val [2]**	HN	7.93
	H^{α}	4.24
	H^{β}	2.08
	H^{γ}	0.91
**Δ^{Z}Phe[3]**	HN	9.79
	H^{β}	7.11
	H^{δ}	7.58
	H^{ε}	7.40
	H^{ζ}	7.48
**Gly [4]**	HN	8.35
	H^{α}	3.96
**ΔAla[5]**	HN	9.27
	$H^{\beta 1}$	5.71

	$H^{\beta2}$	6.21
**OMe**	CH_3	3.77

Table S4: ^{13}C NMR (TMS) of Boc-Gly-**Val**-*Δ^{Z}Phe*-Gly-*ΔAla*-OMe (**2**)

Group	Atom	Chemical shift [ppm]
**Boc**	CH_3	28.63
	C	78.7
	C(O)	156.3
**Gly[1]**	C^{α}	43.6
	C(O)	179.6
**Val [2]**	C^{α}	58.7
	C^{β}	30.4
	C^{γ}	18.6, 19.9
	C(O)	171.7
**Δ^{Z}Phe[3]**	C^{α}	134.1
	C^{β}	128.7
	C^{γ}	128.6
	C^{δ}	130.0
	C^{ε}	130.0
	C^{ζ}	129.6
	C(O)	165.8
**Gly [4]**	C^{α}	43.9
	C(O)	169.0
**ΔAla[5]**	C^{α}	132.9
	C^{β}	109.7
	C(O)	164.2
**OMe**	CH_3	53.1

Table S5: ^{1}H NMR (TMS) of Boc-Gly-*Δ*Ala-Gly-*Δ^{Z}*Phe-Val-OMe (**3**)

Group	Atom	Chemical shift [ppm]
**Boc**	CH_3	1.39
**Gly[1]**	HN	7.22

	H^{α}	3.68
**ΔAla[2]**	HN	9.06
	$H^{\beta 1}$	6.20
	$H^{\beta 2}$	5.60
**Gly [3]**	HN	8.93
	$2H^{\alpha}$	3.94
**Δ^{Z}Phe[4]**	HN	9.59
	H^{β}	7.15
	H^{δ}	7.41
	H^{ε}	7.62
	H^{ζ}	7.35
**Val [5]**	HN	7.97
	H^{α}	4.25
	H^{β}	2.14
	H^{γ}	0.92
**OMe**	CH_3	3.67

Table S6: ^{13}C NMR (TMS) of Boc-Gly-*Δ*Ala-Gly-*Δ*^{Z}Phe-Val-OMe (**3**)

**Group**	**Atom**	**Chemical shift [ppm]**
**Boc**	CH_3	28.6
	C	78.9
	C(O)	156.1
**Gly[1]**	C^{α}	44.8
	C(O)	169.3
**ΔAla[2]**	C^{α}	134.9
	C^{β}	103.7
	C(O)	164.8
**Gly [3]**	C^{α}	43.7
	C(O)	169.1
**Δ^{Z}Phe[4]**	C^{α}	134.2
	C^{β}	130.2
	C^{γ}	130.0
	C^{δ}	129.1
	C^{ε}	130.2

	C^{ζ}	129.3
	C(O)	165.2
**Val [5]**	C^{α}	58.8
	C^{β}	30.4
	C^{γ}	19.4
	C(O)	172.5
**OMe**	CH_3	51.8

Table S7: Interatomic distance restrains for Boc-Val-*Δ*^{Z}Phe-Gly-Gly-*Δ*Ala-OMe (**1**)

	Residue I		Residue II		Distance		
					d-	r_{12}	d+
**Strong**							
	Val[1]	HG#	Val[1]	HB	-0.20	2.00	+0.50
	Gly[3]	HA#	Gly[3]	HN	-0.20	2.00	+0.50
	Gly[4]	HA#	Gly[4]	HN	-0.20	2.00	+0.50
**Medium**							
	Boc	CH_3	Val[1]	HA	-1.20	3.00	+0.50
	Boc	CH_3	Val[1]	HN	-1.20	3.00	+0.50
	Boc	CH_3	Δ^{Z}Phe[2]	HZ	-1.20	3.00	+0.50
	Boc	CH_3	Gly[3]	HA#	-1.20	3.00	+0.50
	Val[1]	HA	Val[1]	HN	-1.20	3.00	+0.50
	Val[1]	HB	Val[1]	HA	-1.20	3.00	+0.50
	Val[1]	HB	Val[1]	HN	-1.20	3.00	+0.50
	Val[1]	HG#	Val[1]	HN	-1.20	3.00	+0.50
	Val[1]	HG#	Val[1]	HA	-1.20	3.00	+0.50
	Val[1]	HG#	Δ^{Z}Phe[2]	HD#	-1.20	3.00	+0.50

	Δ^ZPhe[2]	HB	Δ^ZPhe[2]	HD#	-1.20	3.00	+0.50
	Δ^ZPhe[2]	HB	Gly[3]	HN	-1.20	3.00	+0.50
	Gly[3]	HA#	Gly[4]	HN	-1.20	3.00	+0.50
	Gly[4]	HA#	ΔAla[5]	HN	-1.20	3.00	+0.50
**Weak**							
	Boc	CH_3	Δ^ZPhe[2]	HD#	-2.70	4.50	+0.50
	Boc	CH_3	Gly[3]	HN	-2.70	4.50	+0.50
	Boc	CH_3	ΔAla[5]	HB1	-2.70	4.50	+0.50
	Boc	CH_3	ΔAla[5]	HB2	-2.70	4.50	+0.50
	Boc	CH_3	OMe	CH_3	-2.70	4.50	+0.50
	Val[1]	HA	Δ^ZPhe[2]	HD#	-2.70	4.50	+0.50
	Val[1]	HB	Δ^ZPhe[2]	HD#	-2.70	4.50	+0.50
	Gly[4]	HN	ΔAla[5]	HN	-2.70	4.50	+0.50
	ΔAla[5]	HB1	ΔAla[5]	HN	-2.70	4.50	+0.50
	OMe	CH_3	ΔAla[5]	HB2	-2.70	4.50	+0.50

Table S8: Interatomic distance restrains for Boc-Gly-Val-Δ^ZPhe-Gly-ΔAla-OMe (**2**)

Strong

	Residue I		Residue II		Distance		
					d-	r_{12}	d+
	Boc	CH_3	Val[2]	HB	-0.20	2.00	+0.50
	Gly[1]	HA#	Gly[1]	HN	-0.20	2.00	+0.50
	Gly[1]	HA#	Val[2]	HN	-0.20	2.00	+0.50
	Val[2]	HA	Δ^ZPhe[3]	HN	-0.20	2.00	+0.50
	Val[2]	HG#	Val[2]	HA	-0.20	2.00	+0.50
	Val[2]	HG#	Val[2]	HB	-0.20	2.00	+0.50

	Gly[4]	HA#	Gly[4]	HN	-0.20	2.00	+0.50
**Medium**							
	Val[2]	HA	Val[2]	HN	-1.20	3.00	+0.50
	Val[2]	HB	Val[2]	HA	-1.20	3.00	+0.50
	Val[2]	HB	Val[2]	HN	-1.20	3.00	+0.50
	Val[2]	HB	Δ^ZPhe[3]	HD#	-1.20	3.00	+0.50
	Val[2]	HB	Δ^ZPhe[3]	HN	-1.20	3.00	+0.50
	Val[2]	HG#	Boc	CH_3	-1.20	3.00	+0.50
	Val[2]	HG#	Val[2]	HN	-1.20	3.00	+0.50
	Val[2]	HG#	Δ^ZPhe[3]	HD#	-1.20	3.00	+0.50
	Δ^ZPhe[3]	HB	Δ^ZPhe[3]	HD#	-1.20	3.00	+0.50
	Δ^ZPhe[3]	HB	Gly[4]	HN	-1.20	3.00	+0.50
	Δ^ZPhe[3]	HD#	Δ^ZPhe[3]	HN	-1.20	3.00	+0.50
	Gly[4]	HA#	ΔAla[5]	HN	-1.20	3.00	+0.50
	Gly[4]	HN	Δ^ZPhe[3]	HN	-1.20	3.00	+0.50
**Weak**							
	Boc	CH_3	Gly[1]	HA#	-2.70	4.50	+0.50
	Boc	CH_3	Gly[1]	HN	-2.70	4.50	+0.50
	Boc	CH_3	Vla[2]	HA	-2.70	4.50	+0.50
	Boc	CH_3	Vla[2]	HN	-2.70	4.50	+0.50
	Boc	CH_3	Δ^ZPhe[3]	HB	-2.70	4.50	+0.50
	Boc	CH_3	Δ^ZPhe[3]	HD#	-2.70	4.50	+0.50
	Boc	CH_3	Δ^ZPhe[3]	HE	-2.70	4.50	+0.50
	Boc	CH_3	Δ^ZPhe[3]	HN	-2.70	4.50	+0.50
	Boc	CH_3	Gly[4]	HA#	-2.70	4.50	+0.50
	Boc	CH_3	ΔAla	HB1	-2.70	4.50	+0.50

Boc	CH_3	ΔAla	HB2	-2.70	4.50	+0.50
Boc	CH_3	ΔAla	HN	-2.70	4.50	+0.50
Boc	CH_3	OMe	CH_3	-2.70	4.50	+0.50
Gly[1]	HA#	Val[2]	HA	-2.70	4.50	+0.50
Gly[1]	HA#	Δ^ZPhe[3]	HD#	-2.70	4.50	+0.50
Gly[1]	HA#	Δ^ZPhe[3]	HE#	-2.70	4.50	+0.50
Gly[1]	HA#	Δ^ZPhe[3]	HN	-2.70	4.50	+0.50
Gly[1]	HA#	ΔAla[5]	HB2	-2.70	4.50	+0.50
Gly[1]	HN	Val[2]	HN	-2.70	4.50	+0.50
Val[2]	HA	Δ^ZPhe[3]	HD#	-2.70	4.50	+0.50
Val[2]	HA	Gly[4]	HN	-2.70	4.50	+0.50
Val[2]	HB	Gly[4]	HN	-2.70	4.50	+0.50
Val[2]	HB	Δ^ZPhe[3]	HE#	-2.70	4.50	+0.50
Val[2]	HB	Gly[4]	HN	-2.70	4.50	+0.50
Val[2]	HB	ΔAla[5]	HB2	-2.70	4.50	+0.50
Val[2]	HG#	Gly[1]	HA#	-2.70	4.50	+0.50
Val[2]	HG#	Gly[1]	HN	-2.70	4.50	+0.50
Val[2]	HG#	Δ^ZPhe[3]	HB	-2.70	4.50	+0.50
Val[2]	HG#	Δ^ZPhe[3]	HE#	-2.70	4.50	+0.50
Val[2]	HG#	Δ^ZPhe[3]	HN	-2.70	4.50	+0.50
Val[2]	HG#	Gly[4]	HN	-2.70	4.50	+0.50
Val[2]	HG#	ΔAla[5	HB2	-2.70	4.50	+0.50
Val[2]	HG#	ΔAla[5]	HN	-2.70	4.50	+0.50
Val[2]	HN	Δ^ZPhe[3]	HN	-2.70	4.50	+0.50
Δ^ZPhe[3]	HB	Δ^ZPhe[3]	HN	-2.70	4.50	+0.50
Δ^ZPhe[3]	HB	ΔAla[5]	HN	-2.70	4.50	+0.50

Gly[4]	HA#	ΔAla[5]	HB2	-2.70	4.50	+0.50
Gly[4]	HA#	ΔAla[5]	HN	-2.70	4.50	+0.50
ΔAla[5]	HB1	ΔAla[5]	HN	-2.70	4.50	+0.50
ΔAla[5]	HB2	ΔAla[5]	HN	-2.70	4.50	+0.50
ΔAla[5]	HB1	OMe	CH_3	-2.70	4.50	+0.50

Table S9: Interatomic distance restrains for Boc-Gly-ΔAla-Gly-Δ^ZPhe-Val-OMe (**3**)

Strong

Residue I		Residue II		Distance		
				d-	r_{12}	d+
Boc	CH_3	Gly[1]	HN	-0.20	2.00	+0.50
Boc	CH_3	ΔAla[2]	HN	-0.20	2.00	+0.50
Boc	CH_3	Δ^ZPhe[4]	HD#	-0.20	2.00	+0.50
Boc	CH_3	Δ^ZPhe[4]	HE#	-0.20	2.00	+0.50
Boc	CH_3	Δ^ZPhe[4]	HZ	-0.20	2.00	+0.50
Val[5]	HG#	Val[5]	HA	-0.20	2.00	+0.50
Val[5]	HG#	Val[5]	HB	-0.20	2.00	+0.50

Medium

Boc	CH_3	Gly[1]	HA#	-1.20	3.00	+0.50
Boc	CH_3	Δ^ZPhe[4]	HB	-1.20	3.00	+0.50
Gly[1]	HA#	Gly[1]	HN	-1.20	3.00	+0.50
Gly[1]	HA#	ΔAla[2]	HN	-1.20	3.00	+0.50
Gly[1]	HA#	Val[5]	HA	-1.20	3.00	+0.50
Gly[3]	HA#	Gly[3]	HN	-1.20	3.00	+0.50
Gly[3]	HA#	Δ^ZPhe[4]	HE#	-1.20	3.00	+0.50
Gly[3]	HA#	Δ^ZPhe[4]	HN	-1.20	3.00	+0.50

	Δ^ZPhe[4]	HB	Val[5]	HN	-1.20	3.00	+0.50
	Δ^ZPhe[4]	HE#	Δ^ZPhe[4]	HN	-1.20	3.00	+0.50
	Val[5]	HA	Val[5]	HN	-1.20	3.00	+0.50
	Val[5]	HB	Val[5]	HA	-1.20	3.00	+0.50
	Val[5]	HB	Val[5]	HN	-1.20	3.00	+0.50
	Val[5]	HG#	Gly[1]	HA#	-1.20	3.00	+0.50
	Val[5]	HG#	Gly[3]	HA#	-1.20	3.00	+0.50
	Val[5]	HG#	Val[5]	HN	-1.20	3.00	+0.50
**Weak**							
	Boc	CH_3	ΔAla[2]	HB1	-2.70	4.50	+0.50
	Boc	CH_3	Val[5]	HA	-2.70	4.50	+0.50
	Boc	CH_3	Val[5]	HB	-2.70	4.50	+0.50
	Gly[1]	HA#	ΔAla[3]	HB1	-2.70	4.50	+0.50
	Gly[1]	HA#	Δ^ZPhe[4]	HD#	-2.70	4.50	+0.50
	Gly[1]	HA#	Val[5]	HN	-2.70	4.50	+0.50
	Gly[1]	HN	ΔAla[2]	HN	-2.70	4.50	+0.50
	ΔAla[2]	HB1	ΔAla[2]	HN	-2.70	4.50	+0.50
	ΔAla[2]	HB2	Gly[3]	HN	-2.70	4.50	+0.50
	Gly[3]	HA#	Val[5]	HN	-2.70	4.50	+0.50
	Δ^ZPhe[4]	HB	Δ^ZPhe[4]	HE#	-2.70	4.50	+0.50
	Val[5]	HA	Δ^ZPhe[4]	HB	-2.70	4.50	+0.50
	Val[5]	HA	Δ^ZPhe[4]	HE#	-2.70	4.50	+0.50
	Val[5]	HB	Gly[1]	HA#	-2.70	4.50	+0.50
	Val[5]	HG#	ΔAla[2]	HN	-2.70	4.50	+0.50
	Val[5]	HG#	ΔAla[2]	HB2	-2.70	4.50	+0.50
	Val[5]	HG#	Δ^ZPhe[4]	HB	-2.70	4.50	+0.50

Val[5]	HG#	Δ^{Z}Phe[4]	HE#	-2.70	4.50	+0.50
Val[5]	HG#	Δ^{Z}Phe[4]	HN	-2.70	4.50	+0.50
Val[5]	HN	Δ^{Z}Phe[4]	HN	-2.70	4.50	+0.50

Table S10: Vicinal coupling constants of Val residues from investigated peptides:

Peptide	$^{3}J_{HNHA}$ [Hz]
Boc-Val-Δ^{Z}Phe-Gly-Gly-ΔAla-OMe (**1**)	7.57
Boc-Gly-Val-Δ^{Z}Phe-Gly-ΔAla-OMe (**2**)	7.75
Boc-Gly-ΔAla-Gly-Δ^{Z}Phe-Val-OMe (**3**)	8.41
